# Associations between growth from birth to 18 years, intelligence, and schooling in a Brazilian cohort

**DOI:** 10.1093/ajcn/nqaa047

**Published:** 2020-04-02

**Authors:** Ana Maria Baptista Menezes, Paula D Oliveira, Fernando C Wehrmeister, Luciana Anselmi, Helen Gonçalves, Reynaldo Martorell, Robert E Black, Fernando C Barros, Cesar G Victora

**Affiliations:** Postgraduate Program in Epidemiology, Federal University of Pelotas, Pelotas, Brazil; Postgraduate Program in Epidemiology, Federal University of Pelotas, Pelotas, Brazil; Postgraduate Program in Epidemiology, Federal University of Pelotas, Pelotas, Brazil; Postgraduate Program in Epidemiology, Federal University of Pelotas, Pelotas, Brazil; Postgraduate Program in Epidemiology, Federal University of Pelotas, Pelotas, Brazil; Hubert Department of Global Health, Rollins School of Public Health, Emory University, Atlanta, GA, USA; Department of International Health, Institute for International Programs, Johns Hopkins Bloomberg School of Public Health, Baltimore, MD, USA; Postgraduate Program in Health and Behavior, Catholic University of Pelotas, Pelotas, Brazil; Postgraduate Program in Epidemiology, Federal University of Pelotas, Pelotas, Brazil

**Keywords:** intelligence, schooling, intelligence quotient, conditional growth, linear growth, body mass index, cohort studies

## Abstract

**Background:**

Growth faltering in the first 1000 d is associated with lower human capital among adults. The existence of a second window of opportunity for nutritional interventions during adolescence has been postulated.

**Objectives:**

We aimed to verify the associations between growth from birth to 18 y and intelligence and schooling in a cohort.

**Methods:**

A total of 5249 hospital-born infants in Pelotas, Brazil, were enrolled during 1993. Follow-up visits to random subsamples took place at 6, 12, and 48 mo and to the full cohort at 11, 15, and 18 y. Weight and length/height were collected in all visits. The Wechsler Adult Intelligence Scale was applied at age 18 y, and primary school completion was recorded. Conditional length/height and conditional BMI were calculated and expressed as *z* scores according to the WHO Growth Standards. These express the difference between observed and expected size at a given age based on a regression that includes earlier anthropometric measures. Analyses were adjusted for income, parental education, maternal skin color and smoking, and breastfeeding duration.

**Results:**

In the adjusted analyses, participants with conditional length ≥1 *z* score at 1 y had mean intelligence quotient (IQ) scores at 18 y 4.50 points (95% CI: 1.08, 7.92) higher than those with conditional length ≤−1 at 1 y. For height-for-age at 4 y, this difference was equal to 3.70 (95% CI: 0.49, 6.90) IQ points. There were no associations between conditional height at 11, 15, or 18 y and IQ. For the same previously mentioned comparison, the prevalence ratio for less than primary schooling was 1.42 (95% CI: 1.12, 1.80) for conditional height at 1 y. There were no consistent associations with conditional BMI.

**Conclusions:**

Our findings show that adolescent growth is not associated with intelligence and schooling, and are consistent with the literature on the associations between intelligence and schooling and early linear growth.

## Introduction

In low- and middle-income countries, growth faltering largely takes place in the first 1000 d, from conception to the second birthday (1, 2). Faltering also affects brain development, as the brain reaches ∼55% of its adult size by the age of 2 y, and 90% by the age of 6 y (3, 4). Knowledge about the age range when the brain grows most rapidly is consistent with findings from cohort studies suggesting that linear growth in early childhood is critically important for adult human capital outcomes such as achieved schooling (5, 6) and intelligence (7). These studies also show that linear growth after the first 2 y of life shows little if any association with schooling or intelligence. Early childhood is also a critical time when the benefits of early interventions are amplified and the negative effects of risk factors can be mitigated (8).

Although the major importance of adequate nutrition and intellectual stimulation during the first 1000 d is widely accepted, some authors (9–12) argue that later growth during childhood and adolescence is also important. As summarized by Bundy and Horton (13), “The focus on the first 1,000 days … has caused us to lose sight of the fact that child and adolescent growth and development are complex processes with multiple periods of sensitivity to intervention.”

To understand the relative importance of growth during different age periods on adult human capital, birth cohort studies with measurements throughout childhood and adolescence are needed. Such studies should separate linear growth from relative weight gain, that is, increase in weight above and beyond what is necessary for optimal acquisition of lean mass. We were unable to locate any published studies that have addressed these issues. We report on a birth cohort study from the city of Pelotas in Brazil, in which a population-based sample of children were measured at birth, 1, 4, 11, 15, 18, and 22 y, when schooling and intelligence were also assessed.

## Methods

**FIGURE 1:**
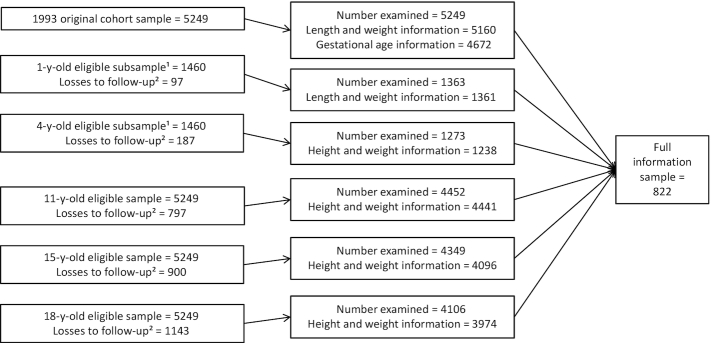
Participant flowchart. Inclusion criteria: participants who had information for weight, length/height, and gestational age. ^1^Follow-up visits including all birth weights <2500 g and a 20% sample of the remaining children. ^2^Includes subjects who either moved out of the study area, could not be located after several attempts, or refused to participate (the latter category included <4% of the sample).

All infants born during the calendar year of 1993 to mothers who lived in the urban area of Pelotas, Brazil, were identified through daily visits to the 5 maternity hospitals in the city. From the 5265 eligible live-born infants, 5249 (99.7%) were enrolled in our birth cohort study. Subsamples of the cohort were followed up at home at ages 6, 12, and 48 mo (14); these included all low-birth-weight children and a random 20% sample of the remaining children, totaling 1460 children. Follow-up rates for this subsample were 96.8% at 6, 93.4% at 12, and 87.2% at 48 mo. At the mean ages of 11, 15, 18, and 22 y, we attempted to contact all cohort members, inviting them to attend the university clinic. In the last wave 4106 participants were interviewed. Those who completed the interviews, added to those known to have died, represent 81.3% of the original cohort. The analyses presented here are restricted to 822 participants who were located in all waves of data collection and who had information for weight, length/height, and gestational age (**Figure 1**); further details on the methodology and participant flowcharts of the 1993 Pelotas (Brazil) birth cohort study are available elsewhere (15).

Birth weight was measured at the hospitals by the study team using pediatric scales (Filizola) with a precision of 10 g, and birth length with a locally made infantometer with a precision of 1 mm. Portable weighing scales were used to measure weight at home visits (PLENNA, HON-00823). Length was measured at 6 and 12 mo with the same infantometer used for the birth measures and standing height was measured at 48 mo. At 11 y, weight was obtained as the mean of 2 measurements using a Tanita digital balance (Tanita UM-080) with accuracy of 100 g. At 15 and 18 y, measurements were taken at the university clinic; the participants were weighed using a portable weighing scale (Tanita UM-080) or a BOD POD scale (COSMED), respectively. For height we used an aluminum stadiometer at all ages. Interviewers were trained and standardized on weight and height measurements before the beginning of fieldwork and every 2 mo afterward to determine the repeatability and validity of the weight and height measurements. Measurements were converted into *z* scores of length- or height-for-age and BMI (in kg/m^2^)-for-age using the WHO Growth Standards (16) for children <5 y, and the WHO Growth Reference for older children and adolescents (17). BMI-for-age was used instead of weight-for-age because the WHO reference curves for the latter end at the age of 10 y.

Two outcome variables were evaluated in the present analysis. Performance in intelligence tests was assessed using the Wechsler Adult Intelligence Scale (third version), which has been adapted to Brazilian samples and used in several Brazilian populations (7, 18–23). The test was administered by 5 trained psychologists at a mean of 18.5 y of age.

Schooling was assessed by whether participants had successfully completed primary schooling or the eighth grade according to the Brazilian school calendar. Primary schooling was used as the outcome based on information collected at 18 y; according to the Brazilian school calendar, the 9 y of primary school should be completed by age 16 y for participants who did not fail any grades. A further visit to the whole cohort took place at the age of 22 y, when schooling information was collected for those who had not been contacted at the age of 18 y. We opted to use primary rather than secondary education as the outcome because at 18 and 22 y some participants were still attending secondary school or secondary-level vocational training.

Conditional growth measures express how an individual child deviates from his or her own previous growth trajectory, also taking into account all other individuals in the cohort. Conditional linear growth includes adjustment for previous length/height-for-age measures, plus adjustment for previous weight-for-age measures. Conditional BMI includes adjustment for previous BMI. Gestational age in weeks was included as a covariate for calculating the scores. Conditional variables are the residuals from linear regressions of anthropometric measures (at a given age) on all prior measures, all of which are expressed as *z* scores based on the WHO standards. For example, a positive residual at 48 mo indicates that a child grew more rapidly in the 12–48 mo age range than was predicted from his/her previous growth, while also considering growth velocity for the whole cohort. In describing the results, we used conditional height or BMI at a given age (e.g., 12 mo) interchangeably with height or BMI gain in the preceding age range (e.g., 6–12 mo). Based on the observed distributions, the following categories were created: ≤−1.00; −0.99 to −0.01; 0.00–0.99; and ≥1.00. The *z* scores at birth were not expressed as conditional variables, given that no earlier measurements were present.

Conditional growth measures have the advantage of assessing growth velocity while taking into account that successive anthropometric measures tend to be correlated. The residuals are independent from previous growth, and are being increasingly used for disentangling the contributions of linear growth and relative weight (a measure of fatness) at different age ranges toward adult outcomes (6, 7, 24, 25).

Confounding variables included family income at birth (minimum wages), parental schooling (in complete years), maternal skin color (white, brown, or black), and maternal smoking during pregnancy (yes/no). Breastfeeding duration was included as a confounder for growth from 4 to 18 y, but not for younger ages in order to avoid reverse causality.

Stata version 12.2 software (Stata Corp.) was used for the analyses. For intelligence quotient (IQ), linear regression was used and results are expressed as differences in IQ points associated with a 1-*z-*score change in the conditional variables. The Wald test for linear trend was used to assess these associations. For primary school completion—a dichotomous variable—we used Poisson regression to report on prevalence ratios.

For both outcomes, we tested for interactions between the conditional growth variables (expressed in continuous *z* scores) and birth weight (<2500 or ≥2500 g), sex (male or female), and parental education (in years of completed schooling, as a continuous variable). Because low-birth-weight children were oversampled in the follow-up visits, statistical weights were included in all regression analyses (including interaction tests) to reproduce the original distribution of birth weight in the full cohort. In the supplementary materials (**Supplemental Tables 1** and **2**) we also used sampling weights to calculate the conditional exposure variables; the results were virtually identical to those presented below in the Results section.

Approval from the Federal University of Pelotas Ethics Committee was obtained for all follow-ups: protocol numbers 158/07, 05/11, and 1.250.366 for the 15-, 18-, and 22-y-old follow-ups, respectively. Written informed consent was obtained from the cohort participants or their caregivers before each phase of the study.

## Results

A total of 822 children had complete data on growth, IQ, and schooling. The baseline characteristics of this subsample were very similar to those of the full cohort (**Table 1**). The main reason for the reduction in the size of the analytical sample was that the measurements of growth during childhood were restricted to a systematic subsample of the cohort, plus all low-birth-weight children. In the full sample, the mean IQ was 97.4 (SE: 0.5) and 71.0% (95% CI: 67.5%, 74.2%) completed 8 y of primary school.

**TABLE 1:** Distribution of baseline characteristics in the full cohort and in the sample included in the present analyses^1^

	Included in analyses^2^ (*n* = 822)	Original cohort (*n* = 5249)
Sex		
Male	48.7 (45.0, 52.4)	49.6 (48.2, 51.0)
Female	51.3 (47.6, 55.0)	50.4 (49.0, 51.8)
Monthly family income, Brazilian minimum wage^3^		
≤1	17.3 (14.5, 20.1)	18.8 (17.8, 19.9)
1.1–3	43.8 (40.1, 47.5)	41.8 (40.5, 43.2)
3.1–6	22.7 (19.6, 25.9)	23.4 (22.3, 24.6)
6.1–10	8.3 (6.2, 10.3)	8.4 (7.7, 9.2)
>10	7.8 (5.8, 9.8)	7.5 (6.8, 8.2)
Maternal education, y		
0–4	24.7 (21.5, 27.9)	28.0 (26.8, 29.2)
5–8	48.8 (45.1, 52.5)	46.2 (44.9, 47.6)
9–11	19.0 (16.1, 21.9)	17.6 (16.6, 18.6)
≥12	7.6 (5.6, 9.6)	8.1 (7.4, 8.9)
Paternal education, y		
0–4	25.1 (21.8, 28.4)	25.6 (24.4, 26.8)
5–8	49.2 (45.4, 53.0)	48.5 (47.1, 49.9)
9–11	18.1 (15.2, 21.1)	18.7 (17.6, 19.8)
≥12	7.6 (5.5, 9.6)	7.3 (6.5, 8.0)
Gestational age, wk		
≤36	9.6 (7.6, 11.6)	11.4 (10.6, 12.4)
37–38	19.6 (16.7, 22.6)	20.0 (18.9, 21.2)
≥39	70.7 (67.4, 72.0)	68.5 (67.2, 69.9)
Birth weight, g		
<2500	8.7 (7.5, 9.9)	9.7 (8.9, 10.6)
2500–2999	21.9 (18.8, 25.1)	25.1 (23.9, 26.2)
3000–3499	43.4 (39.7, 47.1)	39.2 (37.9, 40.5)
≥3500	25.9 (22.6, 29.3)	26.0 (24.8, 27.2)

1 Values are percentages (95% CIs).

2 Because the early follow-up visits included all birth weights < 2500 g and a 20% sample of the remaining children, results were weighted to reproduce the original cohort distribution.

3 Measure of the legal minimum monthly salary for formal employees in Brazil, equivalent to USD 31.4 at the time of enrollment in 1993.

**Supplemental Table 3** shows the distribution of confounding factors and their associations with the intelligence and schooling outcomes. Both outcomes were positively associated with family income at birth and maternal and paternal education, and negatively associated with maternal smoking during pregnancy. IQ was positively associated with birth weight and breastfeeding duration, and gestational age with schooling.

The prevalence of stunting (length- or height-for-age <−2 *z* scores) was 8.1% at age 1 y, 4.7% at age 4 y, 3.8% at age 11 y, 3.5% at age 15 y, and 4.0% at age 18 y. The corresponding proportions of children/adolescents with BMI-for-age below −2 *z* scores were 0.01%, 0.02%, 2.0%, 1.9%, and 1.5% and with BMI > +2 *z* scores were 10.0%, 11.0%, 11.1%, 9.0%, and 10.7%, respectively. The proportion of children born with a weight <2500 g in the cohort was 8.7%.

**Table 2** shows the associations between linear growth and the outcomes. In the crude analyses, conditional lengths (or heights) at birth, 1, and 4 y—but not at later ages—were positively associated with IQ. Controlling for confounding variables reduced the strength of the associations, but these remained significant for ages 1 and 4 y. All associations showed dose-response patterns; the strongest associations were found for conditional length at age 1 y.

**TABLE 2:** IQ and schooling according to conditional length/height-for-age in childhood and adolescence^1^

		Difference in IQ at 18 y, points			Schooling ≥9 complete years at 22 y		
			Regression coefficient			Prevalence ratio	
Length/height-for-age (*z* score)	Frequency, %	Mean (95% CI)	Crude (95% CI) (*n* = 822)	Adjusted (95% CI) (*n* = 773)	% (95% CI)	Crude (95% CI) (*n* = 822)	Adjusted (95% CI) (*n* = 773)
At birth^2^							
≤−1	6.1	93.0 (90.1, 96.0)	Reference (0.00)	Reference (0.00)	60.4 (48.3, 71.3)	Reference (1.00)	Reference (1.00)
−0.99 to −0.01	30.2	96.6 (95.1, 98.1)	3.55 (0.24, 6.86)^4^	1.46 (−2.08, 5.01)	71.2 (64.7, 76.9)	1.18 (0.95, 1.46)	1.08 (0.89, 1.32)
0.00–0.99	45.9	98.0 (96.6, 99.4)	5.00 (1.73, 8.26)^4^	2.27 (−1.22, 5.75)	73.8 (68.4, 78.6)	1.22 (1.00, 1.50)	1.09 (0.89, 1.32)
≥1	17.8	98.7 (96.4, 101.0)	5.68 (1.92, 9.43)^4^	3.31 (−0.73, 7.35)	70.9 (61.6, 78.7)	1.17 (0.93, 1.47)	1.06 (0.86, 1.32)
			*P* = 0.011^4^	*P* = 0.073		*P* = 0.321	*P* = 0.836
At 1 y^3^							
≤−1	13.3	93.7 (91.0, 96.3)	Reference (0.00)	Reference (0.00)	50.0 (39.6, 60.4)	Reference (1.00)	Reference (1.00)
−0.99 to −0.01	35.9	96.0 (94.5, 97.6)	2.37 (−0.72, 5.45)	1.41 (−1.46, 4.28)	70.4 (64.3, 75.8)	1.41 (1.12, 1.76)^4^	1.32 (1.07, 1.65)^4^
0.00–0.99	36.9	98.9 (97.5, 100.3)	5.26 (2.24, 8.28)^4^	3.30 (0.42, 6.18)^4^	76.6 (70.9, 81.5)	1.53 (1.23, 1.91)^4^	1.39 (1.13, 1.72)^4^
≥1	13.9	100.5 (98.3, 102.8)	6.85 (3.37, 10.34)^4^	4.50 (1.08, 7.92)^4^	82.0 (72.9, 88.6)	1.64 (1.30, 2.07)^4^	1.43 (1.15, 1.79)^4^
			*P* < 0.001^4^	*P* = 0.002^4^		*P* < 0.001^4^	*P* = 0.001^4^
At 4 y^3^							
≤−1	15.1	93.8 (91.2, 96.4)	Reference (0.00)	Reference (0.00)	62.1 (52.1, 71.1)	Reference (1.00)	Reference (1.00)
−0.99 to −0.01	36.9	97.1 (95.6, 98.6)	3.28 (0.29, 6.26)^4^	2.78 (−0.19, 5.74)	70.5 (64.4, 75.9)	1.13 (0.95, 1.35)	1.12 (0.95, 1.31)
0.00–0.99	32.4	98.5 (97.0, 100.1)	4.72 (1.71, 7.72)^4^	3.07 (0.14, 6.01)^4^	74.3 (68.2, 79.7)	1.20 (1.01, 1.42)^4^	1.13 (0.96, 1.33)
≥1	15.7	99.4 (97.4, 101.4)	5.58 (2.30, 8.86)^4^	3.70 (0.49, 6.90)^4^	78.1 (69.1, 85.0)	1.26 (1.05, 1.51)^4^	1.20 (1.01, 1.42)^4^
			*P* = 0.004^4^	*P* = 0.032^4^		*P* = 0.008^4^	*P* = 0.048^4^
At 11 y^3^							
≤−1	13.4	97.4 (95.0, 99.7)	Reference (0.00)	Reference (0.00)	75.5 (65.4, 83.3)	Reference (1.00)	Reference (1.00)
−0.99 to −0.01	37.4	98.5 (97.0, 100.1)	1.17 (−1.67, 4.01)	0.84 (−1.96, 3.64)	72.6 (66.8, 77.8)	0.96 (0.84, 1.11)	0.95 (0.83, 1.09)
0.00–0.99	34.4	96.6 (95.1, 98.1)	−0.79 (−4.02, 1.36)	0.00 (−2.80, 2.80)	68.4 (62.0, 74.1)	0.91 (0.78, 1.05)	0.92 (0.80, 1.07)
≥1	14.7	96.5 (94.3, 98.8)	−0.85 (−3.90, 2.35)	0.41 (−2.86, 3.68)	73.6 (63.8, 81.5)	0.98 (0.82, 1.16)	1.00 (0.84, 1.18)
			*P* = 0.290	*P* = 0.860		*P* = 0.470	*P* = 0.837
At 15 y^3^							
≤−1	16.9	97.4 (95.2, 99.7)	Reference (0.00)	Reference (0.00)	78.9 (70.6, 85.3)	Reference (1.00)	Reference (1.00)
−0.99 to −0.01	30.3	98.4 (96.7, 100.0)	0.93 (−1.88, 3.74)	1.14 (−1.45, 3.72)	72.3 (65.8, 78.1)	0.92 (0.81, 1.04)	0.92 (0.81, 1.03)
0.00–0.99	35.3	96.6 (95.1, 98.1)	−0.84 (−3.60, 1.91)	−0.62 (−3.22, 1.99)	69.1 (62.8, 74.7)	0.88 (0.77, 0.99)	0.88 (0.78, 1.00)
≥1	17.6	97.3 (95.2, 99.4)	−0.10 (−3.20, 3.00)	0.92 (−2.01, 3.85)	68.5 (59.4, 76.3)	0.87 (0.74, 1.01)	0.90 (0.78, 1.05)
			*P* = 0.489	*P* = 0.900		*P* = 0.049^4^	*P* = 0.123
At 18 y^3^							
≤−1	14.4	98.1 (95.4, 100.8)	Reference (0.00)	Reference (0.00)	67.0 (57.0, 75.7)	Reference (1.00)	Reference (1.00)
−0.99 to −0.01	36.3	98.4 (97.0, 99.9)	0.34 (−2.72, 3.40)	0.24 (−2.57, 3.04)	73.0 (67.1, 78.2)	1.09 (0.93, 1.28)	1.09 (0.94, 1.27)
0.00–0.99	35.4	96.5 (95.9, 97.9)	−1.65 (−4.71, 1.42)	−0.71 (−3.44, 2.02)	74.8 (68.9, 80.0)	1.12 (0.95, 1.31)	1.16 (1.00, 1.35)
≥1	14.0	96.4 (93.9, 98.9)	−1.70 (−5.40, 2.00)	−1.14 (−4.50, 2.22)	65.2 (55.0, 74.1)	0.97 (0.79, 1.19)	1.01 (0.84, 1.23)
			*P* = 0.218	*P* = 0.311		*P* = 0.986	*P* = 0.490

1 Adjusted for family income at birth, parental years of schooling, maternal skin color, maternal smoking during pregnancy, and total breastfeeding duration. IQ *P* value by Wald's test for linear trend. Schooling *P* value by chi-squared Wald's test. IQ, intelligence quotient (Wechsler Adult Intelligence Scale).

2 Adjusted for the same model, except for total breastfeeding duration.

3 Conditional *z* scores.

4 P values below 0.05 and CIs that do not include the null value.

**Table 3** shows IQ and schooling according to conditional BMI-for-age for the same follow-up visits as in Table 2. In the adjusted analyses, the *P* values for the associations between IQ and BMI at 4 and 11 y were 0.053 and 0.057, respectively. Schooling was associated with BMI at 1 y (*P* = 0.037), but at 11 y the *P* value was 0.052. Unlike what had been observed for conditional length, the associations with BMI did not show clear dose-response patterns.

**TABLE 3:** IQ and schooling according to conditional BMI-for-age in childhood and adolescence^1^

		Difference in IQ at 18 y, points			Schooling ≥9 complete years at 22 y		
			Regression coefficient			Prevalence ratio	
BMI-for-age (*z* score)	Frequency, %	Mean (95% CI)	Crude (95% CI) (*n* = 822)	Adjusted (95% CI) (*n* = 773)	% (95% CI)	Crude (95% CI) (*n* = 822)	Adjusted (95% CI) (*n* = 773)
At birth^2^							
≤−1	8.2	95.9 (93.8, 98.0)	Reference (0.00)	Reference (0.00)	68.8 (58.8, 77.3)	Reference (1.00)	Reference (1.00)
−0.99 to −0.01	29.4	97.1 (95.5, 98.7)	1.25 (−1.40, 3.90)	1.19 (−1.25, 3.63)	68.8 (62.1, 74.8)	1.00 (0.85, 1.18)	1.00 (0.85, 1.16)
0.00–0.99	43.3	98.1 (96.6, 99.6)	2.24 (−0.35, 4.83)	2.04 (−0.35, 4.43)	72.3 (66.6, 77.4)	1.05 (0.90, 1.23)	1.03 (0.89, 1.19)
≥1	19.0	97.0 (95.0, 98.9)	1.09 (−1.75, 3.93)	0.31 (−2.24, 2.85)	75.9 (67.4, 82.8)	1.10 (0.93, 1.31)	1.05 (0.90, 1.23)
			*P* = 0.395	*P* = 0.856		*P* = 0.131	*P* = 0.403
At 1 y^3^							
≤−1	15.2	97.4 (94.8, 99.9)	Reference (0.00)	Reference (0.00)	67.5 (57.7, 76.0)	Reference (1.00)	Reference (1.00)
−0.99 to −0.01	35.6	97.1 (95.6, 98.7)	−0.24 (−3.24, 2.76)	−0.68 (−3.46, 2.09)	69.8 (63.7, 73.4)	1.04 (0.88, 1.21)	1.01 (0.88, 1.18)
0.00–0.99	34.2	97.1 (95.6, 98.5)	−0.29 (−3.24, 2.66)	−0.62 (−3.35, 2.11)	72.6 (66.5, 78.0)	1.08 (0.92, 1.26)	1.08 (0.94, 1.25)
≥1	15.0	98.9 (96.7, 101.1)	1.53 (−1.81, 4.88)	0.90 (−2.37, 4.17)	78.0 (68.9, 85.1)	1.16 (0.97, 1.37)	1.16 (0.98, 1.36)
			*P* = 0.544	*P* = 0.601		*P* = 0.072	*P* = 0.037^4^
At 4 y^3^							
≤−1	12.7	96.8 (94.2, 99.4)	Reference (0.00)	Reference (0.00)	68.4 (57.5, 77.5)	Reference (1.00)	Reference (1.00)
−0.99 to −0.01	44.2	96.5 (95.2, 97.9)	−0.29 (−3.22, 2.65)	−0.72 (−3.59, 2.15)	71.1 (65.7, 75.9)	1.04 (0.88, 1.23)	1.02 (0.88, 1.20)
0.00–0.99	30.5	97.5 (95.7, 99.2)	0.65 (−2.46, 3.77)	0.06 (−2.91, 3.03)	70.6 (63.8, 76.6)	1.03 (0.87, 1.23)	0.99 (0.84, 1.17)
≥1	12.6	101.0 (98.9, 103.2)	4.22 (0.86, 7.58)^4^	2.81 (−0.52, 6.13)	79.4 (69.6, 86.7)	1.16 (0.97, 1.39)	1.12 (0.94, 1.34)
			*P* = 0.005^4^	*P* = 0.053		*P* = 0.158	*P* = 0.343
At 11 y^3^							
≤−1	13.8	96.8 (94.5, 99.1)	Reference (0.00)	Reference (0.00)	71.5 (61.4, 79.8)	Reference (1.00)	Reference (1.00)
−0.99 to −0.01	37.0	96.2 (94.7, 97.8)	−0.58 (−3.36, 2.20)	0.07 (−2.64, 2.78)	65.1 (59.0, 70.7)	0.91 (0.78, 1.07)	0.94 (0.81, 1.09)
0.00–0.99	33.0	98.5 (97.0, 100.1)	1.70 (−1.08, 4.49)	1.86 (−0.85, 4.57)	77.1 (71.1, 82.2)	1.08 (0.93, 1.25)	1.09 (0.94, 1.25)
≥1	15.8	98.3 (96.1, 100.4)	1.43 (−1.74, 4.60)	1.91 (−1.14, 4.96)	75.8 (66.5, 83.2)	1.06 (0.89, 1.26)	1.06 (0.91, 1.24)
			*P* = 0.176	*P* = 0.057		*P* = 0.048^4^	*P* = 0.052
At 15 y^3^							
≤−1	15.3	98.4 (96.1, 100.8)	Reference (0.00)	Reference (0.00)	72.1 (62.6, 80.0)	Reference (1.00)	Reference (1.00)
−0.99 to −0.01	37.2	97.4 (95.9, 98.9)	−1.03 (−3.80, 1.75)	−0.36 (−2.88, 2.16)	75.0 (69.2, 80.0)	1.04 (0.90, 1.20)	1.05 (0.92, 1.20)
0.00–0.99	33.0	97.0 (95.4, 98.6)	−1.46 (−4.31, 1.38)	−1.35 (−4.01, 1.31)	67.2 (60.8, 73.1)	0.93 (0.80, 1.08)	0.94 (0.82, 1.09)
≥1	14.5	97.4 (95.0, 99.8)	−1.03 (−4.40, 2.34)	−0.23 (−3.42, 2.95)	73.2 (63.5, 81.1)	1.01 (0.86, 1.20)	1.03 (0.88, 1.21)
			*P* = 0.795	*P* = 0.570		*P* = 0.479	*P* = 0.575
At 18 y^3^							
≤−1	16.0	98.6 (96.2, 101.0)	Reference (0.00)	Reference (0.00)	66.8 (57.4, 75.1)	Reference (1.00)	Reference (1.00)
−0.99 to −0.01	34.8	96.6 (95.0, 98.2)	−2.03 (−4.92, 0.87)	−1.55 (−4.25, 1.16)	70.3 (64.1, 75.9)	1.05 (0.90, 1.23)	1.05 (0.91, 1.22)
0.00–0.99	33.4	98.4 (96.8, 99.9)	−0.27 (−3.15, 2.61)	−0.56 (−3.30, 2.19)	74.6 (68.5, 80.0)	1.17 (0.96, 1.30)	1.11 (0.96, 1.29)
≥1	15.8	95.9 (94.1, 97.8)	−2.70 (−5.76, 0.35)	−2.51 (−5.42, 0.39)	73.5 (64.6, 80.9)	1.10 (0.92, 1.31)	1.12 (0.96, 1.32)
			*P* = 0.127	*P* = 0.272		*P* = 0.157	*P* = 0.083

1 Adjusted for family income at birth, parental years of schooling, maternal skin color, maternal smoking during pregnancy, and total breastfeeding duration. IQ *P* value by Wald's test for linear trend. Schooling *P* value by chi-squared Wald's test. IQ, intelligence quotient (Wechsler Adult Intelligence Scale).

2 Adjusted for the same model, except for total breastfeeding duration.

3 Conditional *z* scores.

4 P values below 0.05 and CIs that do not include the null value.

We also tested for interactions of conditional growth with birth weight, sex, and maternal education, with IQ as the outcome. **Supplemental Table 4** shows no evidence of effect modification by birth weight or sex in any of the follow-ups. For parental education, only 1 of the 24 interaction terms had *P* < 0.05, which is what one would have expected based on chance alone.

## Discussion

The new, main finding from this analysis was a lack of association between growth during adolescence and the outcomes of IQ at 18 y of age and completion of primary schooling.

In agreement with the existing literature, our analyses confirm the positive association between linear growth in early childhood and both adult intelligence and attained schooling. In contrast, relative changes in BMI in any age period were not consistently or strongly related to intelligence or schooling. Our findings on early growth are well in line with previous results from the COHORTS (Consortium for Health Orientated Research in Transitional Societies) consortium in 5 countries (6) on attained schooling, and with those from the Pelotas 1982 birth cohort on intelligence (7). The 1993 cohort data were not included in either of these previous analyses. The COHORTS and Pelotas 1982 analyses suggest that linear growth from conception to 2 y, but not from 2 to 4 y, is associated with greater intelligence and attained schooling. One limitation of the present analyses is the lack of growth data at the age of 2 y, because cohort members were not visited at this age. Thus, our positive findings regarding growth from 1 to 4 y do not allow separation of these 2 periods.

In addition to COHORTS, the Young Lives Study is an important source of information on growth and human capital. Children from Ethiopia, India, Peru, and Vietnam were followed up from 8 to 15 y of age; those who were stunted at age 8 y and caught up in height by age 15 y had smaller deficits in cognitive scores at that age than did children who remained stunted (26). Because this cohort of children was recruited at 8 y of age, the authors did not account for earlier growth patterns. Other samples of children were followed up at the mean ages of 1–12 y, when reading, vocabulary, and mathematics skills were measured. Length-for-age at 1 y was positively associated with test results at 12 y, and this association was mediated by height-for-age at 5, 8, and 12 y (27). Additional analyses showed that growth from conception through age 1 y, from 1 to 5 y, and from 5 to 8 y, was positively associated with cognitive achievement at age 8 y, again with mediation by attained height at later ages (28). Lastly, after adjustment for length-for-age at 1 y, height-for-age at 8 y was positively associated with attained schooling and mathematics achievement at the age of 8 y (29), but there was no information on heights between 1 and 8 y. None of these analyses, however, covered the whole range of ages from birth to the end of adolescence, nor did they rely on conditional growth modeling to account for the lack of independence of growth in subsequent age ranges. Also, about one-third of all children in this study were stunted at age 8 y, which is well above the prevalence in our cohort. In summary, the Young Lives analyses suggest a positive impact of early growth on development, which is consistent with the present findings, but were unable to quantify the role of growth during several consecutive age ranges as was possible in our analyses.

Our analyses have strengths and limitations. The former include the population-based prospective design with a follow-up rate >80% at the age of 18 y. In addition, the distribution of key characteristics among participants with full data was similar to that in the original cohort (Table 1). Additional strengths include the multiple assessments of growth at different ages with a standardized methodology, and the consistency between the results for intelligence and for schooling, showing that the postulated associations with linear growth were present both for intelligence and for educational achievement, and as a result are also likely to reflect the employment prospects of cohort members. The use of conditional growth analyses represents an advance, because it allowed us to avoid collinearity between successive measurements, as well as to disentangle the roles of linear growth and fatness or relative weight. This approach is increasingly used in the literature (6, 7, 24, 25).

The limitations of our analyses, in addition to the lack of a measurement at the age of 2 y, include the small sample size for identifying interactions with characteristics such as parental education, sex, and birth weight. It is also important to note the relatively low prevalence of stunting or underweight in the sample, which is a limitation when extrapolating our results to other settings where undernutrition is more common. It is possible that variations in growth velocity in our cohort fall mostly within the normal range, unlike what would be the case in high-prevalence populations, but it is important to note that even in a low-prevalence population we were able to detect strong associations between early-life linear growth and intelligence and schooling.

Being solely focused on growth, our analyses do not exclude the possibility that other types of nutrition interventions during adolescence (30)—which do not lead to faster growth—could improve intelligence and attained schooling through other pathways.

Research studies showing the crucial importance of the 1000-d window have been recently criticized for ignoring the possibility of catch-up at later ages, with potential implications for adult human capital (13). In several ways, this is a false dichotomy. Interventions that promote linear growth during the first 1000 d are aimed at preventing the occurrence of stunting, rather than attempting to recover older children who are already stunted. Brain growth largely takes place in this early critical window, when the acquisition of cognitive, language, and socioemotional skills is fastest (31). The fact that such brain functions and associated skills continue to develop throughout late childhood and adolescence, and that cognitive and nutritional interventions may result in improved adult outcomes (9), does not detract from the potentially greater impact of early interventions. In addition, interventions for promoting human capital such as breastfeeding promotion are limited to the first couple of years of life (32, 33).

Even though improvements in child growth after early faltering may bring benefits to acquisition of human capital, it remains to be shown that interventions that are effective do not contribute to excessive adolescent weight gain, and that the cost–benefit of such interventions is of similar magnitude to that of interventions aimed at preventing, rather than alleviating, growth faltering.

## Supplementary Material

nqaa047_Supplement_File

## Notes

Supported by the Science and Technology Department, Brazilian Ministry of Health, with resources transferred through Brazilian National Council for Scientific and Technological Development (CNPq) grant 400943/2013–1 and financed in part by the Coordenação de Aperfeiçoamento de Pessoal de Nível Superior—Brasil— Finance Code 001. The study “Pelotas Birth Cohort, 1993” was conducted by the Postgraduate Program in Epidemiology at Universidade Federal de Pelotas, with the collaboration of the Brazilian Public Health Association (ABRASCO). From 2004 to 2013, Wellcome Trust grant 086974/Z/08/Z supported the 1993 Birth Cohort study. The initial phases of the cohort were funded by the European Union and the Brazilian National Program for Centers of Excellence (PRONEX), the National Research Council (CNPq), and the Ministry of Health.

The funders did not have any role in the design, data collection, analysis, or interpretation of the study findings.

Supplemental Tables 1–4 are available from the “Supplementary data” link in the online posting of the article and from the same link in the online table of contents at https://academic.oup.com/ajcn.
