# Supplementary material for: Associations between growth from birth to 18 years, intelligence, and schooling in a Brazilian cohort
Source: Am J Clin Nutr. 2020 Apr 2;112(1):187–94. doi: 10.1093/ajcn/nqaa047 (PMC7326584; doi:10.1093/ajcn/nqaa047)
Supplement: nqaa047_Supplement_File [file nqaa047_supplement_file.docx]

**Associations between growth from birth to 18 years, intelligence and schooling in a Brazilian cohort**

**Ana Maria B. Menezes, et al.**

**Supplementary Table 1. IQ and schooling according to conditional length/height for age (weighted variables) in childhood and adolescence. P values below 0.05 and confidence intervals that do not include the null value are shown in bold font.**

|  | Frequency | Difference of IQ (points) at 18 years | | | Schooling ≥ 9 complete years at 22 years | | |
| --- | --- | --- | --- | --- | --- | --- | --- |
| **Length/height for age (z score)** | % | Mean (95% CI) | Regression Coefficient | | % (95% CI) | Prevalence Ratio | |
|  |  |  | Crude (95% CI)  n= 822 | Adjusted (95% CI)  n= 773 |  | Crude (95% CI)  n= 822 | Adjusted (95% CI)  n= 773 |
| **At birth^1^** |  |  |  |  |  |  |  |
| ≤−1 | 6.1 | 92.8 (89.9, 95.8) | Reference (0) | Reference (0) | 58.9 (47.4, 70.4) | Reference (1) | Reference (1) |
| −0.99 to −0.01 | 28.3 | 96.5 (95.0, 98.0) | **3.71 (0.40, 7.01)** | 1.83 (-1.71, 5.37) | 68.7 (62.5, 74.9) | 1.17 (0.94, 1.45) | 1.04 (0.86, 1.27) |
| 0.00 to 0.99 | 47.8 | 98.0 (96.6, 99.4) | **5.18 (1.94, 8.42)** | 2.27 (-1.21, 5.74) | 73.7 (68.8, 78.6) | **1.25 (1.01, 1.53)** | 1.07 (0.89, 1.29) |
| ≥1 | 17.8 | 98.8 (96.4, 101.1) | **5.94 (2.20, 9.67)** | 3.12 (-0.83, 7.08) | 71.6 (63.3, 79.8) | 1.22 (0.97, 1.52) | 1.03 (0.83, 1.26) |
|  |  |  | **p= 0.006** | p= 0.141 |  | p= 0.153 | p= 0.862 |
| **At 1 year²** |  |  |  |  |  |  |  |
| ≤−1 | 13.0 | 93.9 (91.2, 96.6) | Reference (0) | Reference (0) | 50.9 (40.7, 61.1) | Reference (1) | Reference (1) |
| −0.99 to −0.01 | 36.7 | 96.1 (94.6, 97.6) | 2.21 (-0.93, 5.35) | 1.35 (-1.56, 4.26) | 69.9 (64.4, 75.5) | **1.37 (1.11, 1.71)** | **1.37 (1.10, 1.71)** |
| 0.00 to 0.99 | 37.5 | 98.9 (97.4, 100.3) | **4.96 (1.87, 8.06)** | **2.95 (0.02, 5.88)** | 76.4 (71.2, 81.5) | **1.50 (1.21, 1.85)** | **1.41 (1.13, 1.75)** |
| ≥1 | 12.7 | 100.5 (98.2, 102.8) | **6.59 (3.03, 10.16)** | **4.51 (1.00, 8.01)** | 78.9 (70.4, 87.4) | **1.55 (1.23, 1.95)** | **1.42 (1.12, 1.80)** |
|  |  |  | **p< 0.001** | **p= 0.004** |  | **p= 0.001** | **p= 0.003** |
| **At 4 years²** |  |  |  |  |  |  |  |
| ≤−1 | 15.4 | 93.6 (91.0, 96.1) | Reference (0) | Reference (0) | 61.1 (51.8, 70.4) | Reference (1) | Reference (1) |
| −0.99 to −0.01 | 37.2 | 97.0 (95.6, 98.5) | **3.49 (0.53, 6.45)** | **3.56 (0.65, 6.46)** | 69.3 (63.7, 74.9) | 1.13 (0.95, 1.35) | 1.15 (0.98, 1.35) |
| 0.00 to 0.99 | 33.5 | 98.6 (97.1, 100.1) | **5.06 (2.08, 8.03)** | **3.76 (0.90, 6.62)** | 74.7 (69.2, 80.1) | **1.22 (1.03, 1.45)** | 1.15 (0.98, 1.34) |
| ≥1 | 13.9 | 99.7 (97.6, 101.9) | **6.16 (2.82, 9.50)** | **4.72 (1.50, 7.94)** | 78.7 (69.6, 85.8) | **1.27 (1.06, 1.53)** | **1.20 (1.01, 1.43)** |
|  |  |  | **p= 0.001** | **p= 0.007** |  | **p= 0.039** | p= 0.069 |
| **At 11 years²** |  |  |  |  |  |  |  |
| ≤−1 | 13.6 | 97.5 (95.1, 99.7) | Reference (0) | Reference (0) | 77.1 (68.7, 85.5) | Reference (1) | Reference (1) |
| −0.99 to −0.01 | 37.3 | 98.4 (96.9, 100.1) | 0.92 (-1.88, 3.74) | 0.66 (-2.08, 3.41) | 71.2 (65.7, 76.6) | 0.92 (0.81, 1.05) | 0.93 (0.82, 1.07) |
| 0.00 to 0.99 | 34.1 | 96.8 (95.2, 98.1) | -0.71 (-3.52, 2.10) | 0.14 (-2.61, 2.89) | 68.2 (62.3, 74.1) | 0.88 (0.77, 1.02) | 0.92 (0.80, 1.06) |
| ≥1 | 15.0 | 96.3 (94.1, 98.6) | -1.13 (-4.39, 2.12) | 0.34 (-2.90, 3.58) | 71.4 (63.7, 80.0) | 0.92 (0.79, 1.09) | 0.99 (0.84, 1.16) |
|  |  |  | p= 0.370 | p= 0.951 |  | p= 0.390 | p= 0.814 |
| **At 15 years²** |  |  |  |  |  |  |  |
| ≤−1 | 16.9 | 97.3 (95.1, 99.6) | Reference (0) | Reference (0) | 79.1 (71.9, 86.3) | Reference (1) | Reference (1) |
| −0.99 to −0.01 | 30.1 | 98.7 (97.1, 100.4) | 1.42 (-1.32, 4.17) | 1.44 (-1.12, 4.00) | 73.2 (67.3, 79.2) | 0.93 (0.82, 1.04) | 0.92 (0.82, 1.04) |
| 0.00 to 0.99 | 35.4 | 96.3 (94.7, 97.8) | -1.05 (-3.77, 1.67) | -0.62 (-3.19, 1.94) | 66.7 (60.9, 74.6) | 0.84 (0.74, 0.96) | 0.88 (0.78, 0.99) |
| ≥1 | 17.6 | 97.4 (95.3, 99.5) | 0.09 (-2.97, 3.14) | 1.16 (-1.73, 4.05) | 68.0 (59.8, 76.2) | 0.86 (0.74, 1.00) | 0.90 (0.77, 1.05) |
|  |  |  | p= 0.199 | p= 0.920 |  | **p= 0.045** | p= 0.102 |
| **At 18 years²** |  |  |  |  |  |  |  |
| ≤−1 | 14.5 | 98.4 (95.8, 101.0) | Reference (0) | Reference (0) | 64.8 (55.4, 74.2) | Reference (1) | Reference (1) |
| −0.99 to −0.01 | 38.2 | 98.3 (96.9, 99.7) | -0.06 (-3.02, 2.91) | 0.24 (-2.53, 3.01) | 73.6 (68.4, 78.8) | 1.14 (0.97, 1.33) | 1.14 (0.98, 1.33) |
| 0.00 to 0.99 | 32.9 | 96.4 (94.9, 97.9) | -1.97 (-4.98, 1.04) | -0.62 (-3.34, 2.11) | 72.9 (67.2, 78.6) | 1.13 (0.95, 1.33) | 1.21 (1.05, 1.41) |
| ≥1 | 14.4 | 96.4 (93.9, 98.9) | -1.97 (-5.56, 1.63) | -1.00 (-4.30, 2.30) | 66.0 (55.8, 75.1) | 1.02 (0.83, 1.24) | 1.06 (0.88, 1.29) |
|  |  |  | p= 0.227 | p= 0.365 |  | p= 0.266 | p= 0.286 |

Adjusted for family income at birth, parental years of schooling, maternal skin color, maternal smoking during pregnancy and total breastfeeding duration.^1^Adjusted for the same model, except breastfeeding. ²Conditional z scores IQ *P*-value by Wald’s test for linear trend. Schooling *P-value* by chi-squared Wald’s test. IQ: Intelligence quotient (Wechsler Adult Intelligence Scale). CI: confidence interval.

**Supplementary Table 2. IQ and schooling at 18 years according to conditional BMI for age (weighted variables) in childhood and adolescence. P values below 0.05 and confidence intervals that do not include the null value are shown in bold font.**

|  | Frequency | Difference of IQ (points) at 18 years | | | Schooling ≥ 9 complete years at 22 years | | |
| --- | --- | --- | --- | --- | --- | --- | --- |
| **BMI for age (*z* score)** | % | Mean (95% CI) | Regression Coefficient | | % (95% CI) | Prevalence Ratio | |
|  |  |  | Crude (95% CI)  n= 822 | Adjusted (95% CI)  n= 773 |  | Crude (95% CI)  n= 822 | Adjusted (95% CI)  n= 773 |
| **At birth^1^** |  |  |  |  |  |  |  |
| ≤−1 | 7.4 | 95.2 (93.1, 97.3) | Reference (0) | Reference (0) | 66.1 (56.5, 75.8) | Reference (1) | Reference (1) |
| −0.99 to −0.01 | 27.6 | 97.5 (95.8, 99.2) | 2.33 (-0.37, 5.02) | 1.99 (-0.43, 4.42) | 70.0 (63.7, 76.3) | 1.06 (0.89, 1.26) | 1.07 (0.91, 1.25) |
| 0.00 to 0.99 | 46.9 | 97.8 (96.3, 99.2) | **2.57 (0.03, 5.11)** | **2.27 (0.01, 4.52)** | 71.2 (66.1, 76.2) | 1.08 (0.92, 1.26) | 1.09 (0.93, 1.26) |
| ≥1 | 18.1 | 97.2 (95.2, 98.1) | 1.96 (-0.93, 4.85) | 0.67 (-1.85, 3.18) | 74.1 (66.2, 82.0) | 1.12 (0.94, 1.34) | 1.11 (0.94, 1.31) |
|  |  |  | p= 0.236 | p= 0.997 |  | p= 0.653 | p= 0.259 |
| **At 1 year²** |  |  |  |  |  |  |  |
| ≤−1 | 15.3 | 97.5 (95.0, 100.1) | Reference (0) | Reference (0) | 67.5 (58.6, 76.4) | Reference (1) | Reference (1) |
| −0.99 to −0.01 | 35.4 | 97.1 (95.6, 98.7) | -0.39 (-3.40, 2.61) | -0.41 (-3.20, 2.38) | 69.4 (63.7, 75.2) | 1.03 (0.88, 1.20) | 1.02 (0.88, 1.18) |
| 0.00 to 0.99 | 34.4 | 97.1 (95.6, 98.5) | -0.44 (-3.40, 2.52) | -0.42 (-3.15, 2.32) | 71.3 (65.6, 77.0) | 1.06 (0.92, 1.23) | 1.09 (0.94, 1.26) |
| ≥1 | 14.9 | 98.7 (96.6, 100.9) | 1.23 (-2.14, 4.60) | 0.86 (-2.44, 4.16) | 77.6 (69.7, 85.5) | 1.15 (0.97, 1.36) | 1.15 (0.98, 1.35) |
|  |  |  | p= 0.623 | p= 0.647 |  | p= 0.298 | **p= 0.041** |
| **At 4 years²** |  |  |  |  |  |  |  |
| ≤−1 | 12.7 | 97.0 (94.5, 99.5) | Reference (0) | Reference (0) | 69.2 (59.6, 79.0) | Reference (1) | Reference (1) |
| −0.99 to −0.01 | 44.7 | 96.5 (95.1, 97.8) | -0.51 (-3.39, 2.36) | -0.75 (-3.57, 2.06) | 70.9 (65.8, 75.9) | 1.02 (0.87, 1.20) | 1.01 (0.87, 1.17) |
| 0.00 to 0.99 | 30.7 | 97.5 (95.8, 99.2) | 0.51 (-2.55, 3.37) | 0.02 (-2.88, 2.92) | 68.5 (62.3, 74.7) | 0.99 (0.84, 1.17) | 0.96 (0.82, 1.13) |
| ≥1 | 12.9 | 100.8 (98.7, 102.9) | **3.86 (0.58, 7.14)** | 2.53 (-0.71, 5.77) | 79.2 (70.9, 87.4) | 1.14 (0.96, 1.36) | 1.11 (0.94, 1.31) |
|  |  |  | **p= 0.007** | p= 0.070 |  | p= 0.190 | p= 0.465 |
| **At 11 years²** |  |  |  |  |  |  |  |
| ≤−1 | 13.6 | 96.7 (94.4, 99.0) | Reference (0) | Reference (0) | 73.6 (64.9, 82.3) | Reference (1) | Reference (1) |
| −0.99 to −0.01 | 37.6 | 96.5 (94.9, 98.0) | -0.25 (-3.02, 2.52) | 0.39 (-2.30, 3.07) | 65.3 (59.6, 71.0) | 0.89 (0.77, 1.03) | 0.93 (0.81, 1.08) |
| 0.00 to 0.99 | 32.0 | 98.3 (97.7, 99.8) | 1.56 (-1.21, 4.33) | 1.83 (-0.84, 4.49) | 75.7 (70.0, 81.3) | 1.03 (0.89, 1.18) | 1.09 (0.95, 1.25) |
| ≥1 | 16.8 | 98.5 (96.3, 100.6) | 1.76 (-1.37, 4.89) | 1.74 (-1.24, 4.73) | 72.7 (64.7, 80.9) | 0.99 (0.84, 1.16) | 1.03 (0.88, 1.21) |
|  |  |  | p= 0.277 | p= 0.099 |  | p= 0.083 | p= 0.098 |
| **At 15 years²** |  |  |  |  |  |  |  |
| ≤−1 | 14.4 | 98.5 (96.0, 100.9) | Reference (0) | Reference (0) | 69.9 (60.9, 78.9) | Reference (1) | Reference (1) |
| −0.99 to −0.01 | 38.2 | 97.4 (95.9, 98.9) | -1.06 (-3.91, 1.79) | -0.70 (-3.32, 1.92) | 74.7 (69.5, 79.9) | 1.07 (0.92, 1.24) | 1.05 (0.92, 1.20) |
| 0.00 to 0.99 | 32.8 | 96.9 (95.3, 98.5) | -1.58 (-4.51, 1.34) | -1.56 (-4.31, 1.19) | 67.1 (61.1, 73.1) | 0.96 (0.82, 1.12) | 0.94 (0.81, 1.08) |
| ≥1 | 14.6 | 97.6 (95.2, 99.9) | -0.89 (-4.31, 2.53) | -0.42 (-3.68, 2.85) | 71.1 (62.3, 79.8) | 1.06 (0.85, 1.21) | 1.05 (0.88, 1.23) |
|  |  |  | p= 0.754 | p= 0.556 |  | p= 0.307 | p= 0.649 |
| **At 18 years²** |  |  |  |  |  |  |  |
| ≤−1 | 16.1 | 98.6 (96.2, 101.0) | Reference (0) | Reference (0) | 66.7 (57.9, 75.4) | Reference (1) | Reference (1) |
| −0.99 to −0.01 | 35.2 | 96.8 (95.2, 98.4) | -1.87 (-4.75, 1.01) | -1.33 (-4.04, 1.38) | 68.5 (62.7, 74.3) | 1.03 (0.88, 1.20) | 1.08 (0.93, 1.26) |
| 0.00 to 0.99 | 33.6 | 98.2 (96.6, 99.8) | -0.44 (-3.31, 2.43) | -0.66 (-3.41, 2.10) | 75.5 (70.1, 81.0) | 1.13 (0.98, 1.32) | 1.15 (1.00, 1.34) |
| ≥1 | 15.2 | 95.8 (93.9, 97.7) | -2.81 (-5.86, 0.25) | -2.55 (-5.49, 0.38) | 71.4 (63.1, 79.7) | 1.07 (0.90, 1.28) | 1.11 (0.94, 1.32) |
|  |  |  | p= 0.158 | p= 0.220 |  | p= 0.230 | p= 0.100 |

Adjusted for family income at birth, parental years of schooling, maternal skin color, maternal smoking during pregnancy and total breastfeeding duration. ^1^Adjusted for the same model, except breastfeeding. ²Conditional z scores. IQ *P*-value by Wald’s test for linear trend. Schooling *P-value* by chi-squared Wald’s test. IQ: Intelligence quotient (Wechsler Adult Intelligence Scale). CI: confidence interval.

**Supplementary Table 3. Characteristics of study sample, intelligence quotient (IQ) and years of schooling at 22 years according to covariates (n=822).**

|  | **Frequency** | **IQ (points)** | **Schooling**  **(≥ 9 years)** |
| --- | --- | --- | --- |
| **Variables** | **%** | **Mean (95% CI)** | **% (95% CI)** |
| **Monthly family income (minimum wage)** |  |  |  |
| ≤ 1 | 17.3 | 90.5 ( 88.3, 92.7) | 43.3 (34.8, 52.2) |
| 1.1 – 3 | 43.8 | 97.1 (95.8, 98.4) | 69.3 (64.0, 74.3) |
| 3.1 – 6 | 22.8 | 98.8 (97.2, 100.5) | 80.4 (73.6, 85.9) |
| 6.1 – 10 | 8.3 | 102.3 (99.5, 105.1) | 88.8 (78.1, 94.6) |
| > 10 | 7.8 | 105.6 (101.9, 109.3) | 96.0 (85.4, 99.0) |
|  |  | p< 0.001 | p< 0.001 |
| **Maternal education (years)** |  |  |  |
| 0 – 4 | 24.8 | 93.1 (91.4, 94.8) | 56.8 (49.3, 63.9) |
| 5 – 8 | 48.8 | 96.1 (94.8, 97.3) | 67.1 (61.9, 71.9)) |
| 9 – 11 | 19.0 | 102.4 (100.5, 104.3) | 90.7 (84.4, 94.5) |
| ≥ 12 | 7.6 | 107.8 (104.6, 111.0) | 93.9 (82.7, 98.0) |
|  |  | p< 0.001 | p< 0.001 |
| **Paternal education (years)** |  |  |  |
| 0 – 4 | 25.1 | 94.3 (92.5, 96.0) | 53.2 (45.5, 60.6) |
| 5 – 8 | 49.2 | 96.6 (95.4, 97.9) | 71.6 (66.5, 76.2) |
| 9 - 11 | 18.1 | 102.0 (99.8, 104.2) | 88.6 (81.6, 93.1) |
| ≥ 12 | 7.6 | 106.4 (103.2, 109.6) | 93.5 (81.8, 97.9) |
|  |  | p< 0.001 | p< 0.001 |
| **Gestational age (weeks)** |  |  |  |
| ≤ 36 | 9.6 | 95.5 (92.5, 98.5) | 54.2 (43.5, 64.4) |
| 37-38 | 19.6 | 96.5 (94.5, 98.5) | 64.5 (56.1, 72.0) |
| ≥39 | 70.7 | 97.9 (96.8, 99.0) | 75.1 (71.1, 78.8) |
|  |  | p= 0.202 | p< 0.001 |
| **Birthweight (grams)** |  |  |  |
| < 2500 | 8.7 | 94.5 (92.9, 96.1) | 59.5 (52.9, 65.7) |
| 2500 - 2999 | 21.9 | 97.7 (95.7,99.7) | 69.9 (61.9, 76.9) |
| 3000 - 3499 | 43.4 | 96.9 (95.5, 98.3) | 71.4 (65.8, 76.4) |
| ≥3500 | 25.9 | 99.0 (97.1, 100.9) | 75.1 (68.1, 81.1) |
|  |  | p= 0.003 | P=0.094 |
| **Maternal smoking during pregnancy** |  |  |  |
| No | 70.1 | 98.4 (97.4, 99.5) | 74.9 (70.8, 78.5) |
| Yes | 29.9 | 94.9 (93.3, 96.6) | 61.9 (55.2, 68.1) |
|  |  | p= 0.001^1^ | p< 0.001 |
| **Breastfeeding duration (months)** |  |  |  |
| < 1 | 14.0 | 95.5 (93.2, 97.8) | 74.7 (65.5, 82.2) |
| 1-2.9 | 24.3 | 95.5 (93.9, 97.1) | 66.2 (58.8, 72.9) |
| 3-5.9 | 24.7 | 97.6 (95.7, 99.5) | 71.3 (64.2, 77.5) |
| ≥6 | 37.0 | 99.3 (97.7, 100.8) | 72.5 (66.7, 77.6) |
|  |  | p= 0.004 | p= 0.410 |
| **Total** | 822 | 97.4 (96.5, 98.3) | 71.0 (67.5, 74.2) |

P-level IQ: ANOVA for linear trend, except ^1^p-level (ANOVA). Schooling: qui-squared test**.** IQ: Wechsler Adult Intelligence Scale.

**Supplementary Table 4. Conditional length/height/BMI for age analyses of intelligence quotient (IQ) at 18 years according to birthweight, sex and parental education.^1^**

|  | **Adjusted regression coefficient (95% CI)^2^** | | | ***P*-value for interaction**  **(continuous)** | **P-value for interaction**  **(dichotomous birthweight)** |
| --- | --- | --- | --- | --- | --- |
|  | **Birthweight** | | |  |  |
|  | **<2500 grams (n= 216)** | | **≥2500 grams (n= 557)** |  |  |
| **Length/height for age (z score)** |  |  | |  |  |
| At birth | 0.93 (-0.59, 2.46) | 0.74 (-0.62, 2.10) | | 0.218 | 0.910 |
| At 1 year³ | 1.19 (-0.10, 2.48) | 1.94 (0.90, 2.97) | | 0.586 | 0.824 |
| At 4 years³ | 0.70 (-0.68, 2.09) | 1.12 (0.07, 2.17) | | 0.550 | 0.965 |
| At 11 years³ | 1.48 (0.09, 2.86) | 0.09 (-0.89, 1.08) | | 0.673 | 0.474 |
| At 15 years³ | 0.00 (-1.24, 1.23) | 0.05 (-0.94, 1.05) | | 0.228 | 0.872 |
| At 18 years³ | 0.49 (-0.96, 1.93) | -0.42 (-1.41, 0.58) | | 0.170 | 0.436 |
| **BMI for age (*z* score)** |  |  | |  |  |
| At birth | 1.05 (-0.21, 2.30) | -0.35 (-1.42, 0.72) | | 0.121 | 0.202 |
| At 1 year³ | 1.47 (0.06, 2.89) | -0.06 (-1.07, 0.95) | | 0.179 | 0.058 |
| At 4 years³ | -0.10 (-1.60, 1.41) | 1.24 (0.36, 2.12) | | 0.129 | 0.239 |
| At 11 years³ | 0.57 (-0.87, 2.00) | 0.57 (-0.39, 1.53) | | 0.118 | 0.885 |
| At 15 years³ | -0.25 (-1.76, 1.26) | -0.14 (-1.02, 0.74) | | 0.926 | 0.899 |
| At 18 years³ | 0.50 (-0.75, 1.74) | -0.59 (-1.50, 0.32) | | 0.348 | 0.202 |
|  | **Sex** | | | ***P*-value for interaction** | **_** |
|  | **Males (n= 370)** | **Females (n= 403)** | |  |  |
| **Length/height for age (z score)** |  |  | |  |  |
| At birth | 1.86 (0.30, 3.43) | 0.10 (-1.15, 1.37) | | 0.094 | - |
| At 1 year³ | 2.37 (1.12, 3.63) | 1.41 (0.04, 2.79) | | 0.497 | - |
| At 4 years³ | 1.15 (-0.10, 2.40) | 0.92 (-0.45, 0.24) | | 0.719 | - |
| At 11 years³ | -0.68 (-2.28, 0.93) | 0.74 (-0.33, 1.81) | | 0.167 | - |
| At 15 years³ | 0.27 (-1.08, 1.62) | -0.59 (-1.94, 0.76) | | 0.386 | - |
| At 18 years³ | -0.28 (-1.37, 0.79) | -0.31 (-2.11, 1.47) | | 0.905 | - |
| **BMI for age (*z* score)** |  |  | |  |  |
| At birth | 2.19 (-1.50, 5.87) | -1.30 (-5.51, 2.91) | | 0.682 | - |
| At 1 year³ | 0.05 (-1.24, 1.34) | 0.09 (-1.19, 1.37) | | 0.945 | - |
| At 4 years³ | 1.22 (0.02, 2.42) | 1.07 (-0.06, 2.20) | | 0.688 | - |
| At 11 years³ | 1.31 (0.01, 2.61) | -0.39 (-1.55, 0.77) | | 0.051 | - |
| At 15 years³ | -0.68 (-1.93, 0.56) | 0.15 (-0.94, 1.25) | | 0.274 | - |
| At 18 years³ | -1.18 (-2.46, 0.10) | 0.36 (-0.70, 1.43) | | 0.077 | - |
|  | **Maternal education** | | | ***P*-value for interaction**  **(continuous)** | ***P*-value for interaction**  **(dichotomous maternal education)** |
|  | **<6 years (n= 331)** | **>=6 years (n= 442)** | |  |  |
| **Length/height for age (z score)** |  |  | |  |  |
| At birth | 0.50 (-1.22, 2.21) | 1.22 (-0.07, 2.51) | | 0.585 | 0.550 |
| At 1 year³ | 2.35 (0.86, 3.84) | 1.55 (0.36, 2.74) | | 0.150 | 0.562 |
| At 4 years³ | 0.67 (-0.70, 2.04) | 1.44 (0.17, 2.71) | | 0.290 | 0.383 |
| At 11 years³ | -0.45 (-1.85, 0.96) | 0.48 (-0.67, 1.64) | | 0.450 | 0.443 |
| At 15 years³ | 0.40 (-1.06, 1.85) | -0.15 (-1.34, 1.02) | | 0.510 | 0.606 |
| At 18 years³ | 0.00 (-1.54, 1.53) | -0.59 (-1.69, 0.50) | | 0.697 | 0.508 |
| **BMI for age (*z* score)** |  |  | |  |  |
| At birth | 0.69 (-0.64, 2.01) | -0.27 (-1.36, 0.81) | | 0.495 | 0.248 |
| At 1 year³ | 0.65 (-0.77, 2.08) | -0.22 (-1.41, 0.98) | | 0.649 | 0.480 |
| At 4 years³ | 1.76 (0.27, 3.25) | 0.77 (-0.21, 1.75) | | 0.899 | 0.278 |
| At 11 years³ | 1.00 (-0.36, 2.34) | 0.32 (-0.85, 1.49) | | 0.470 | 0.362 |
| At 15 years³ | -0.17 (-1.48, 1.13) | -0.18 (-1.26, 0.89) | | 0.287 | 0.996 |
| At 18 years³ | -0.48 (-1.82, 0.86) | -0.55 (-1.63, 0.54) | | 0.247 | 0.804 |
|  | **Paternal education** | | | ***P*-value for interaction**  **(continuous)** | ***P*-value for interaction**  **(dichotomous paternal education)** |
|  | **<6 years (n= 329)** | **>=6 years (n= 444)** | |  |  |
| **Length/height for age (z score)** |  |  | |  |  |
| At birth | 1.41 (-0.30, 3.12) | 0.61 (-0.69, 1.92) | | 0.140 | 0.385 |
| At 1 year³ | 2.56 (1.15, 3.96) | 1.37 (0.13, 2.61) | | 0.050 | 0.180 |
| At 4 years³ | 1.18 (-0.18, 2.54) | 1.04 (-0.25, 2.32) | | 0.821 | 0.778 |
| At 11 years³ | -0.35 (-1.68, 0.98) | 0.47 (-0.73, 1.66) | | 0.774 | 0.343 |
| At 15 years³ | -0.12 (-1.47, 1.22) | 0.28 (-0.99, 1.55) | | 0.507 | 0.551 |
| At 18 years³ | -0.61 (-2.05, 0.84) | -0.03 (-1.25, 1.18) | | 0.294 | 0.544 |
| **BMI for age (*z* score)** |  |  | |  |  |
| At birth | 0.64 (-0.74, 2.01) | -0.17 (-1.24, 0.90) | | 0.615 | 0.289 |
| At 1 year³ | 0.61 (-0.82, 2.03) | -0.23 (-1.42, 0.95) | | 0.563 | 0.438 |
| At 4 years³ | 1.25 (0.04, 2.46) | 1.04 (-0.09, 2.16) | | 0.838 | 0.755 |
| At 11 years³ | 1.22 (-0.22, 2.68) | 0.37 (-0.77, 1.50) | | 0.022 | 0.400 |
| At 15 years³ | 0.17 (-1.16, 1.51) | -0.36 (-1.40, 0.70) | | 0.211 | 0.597 |
| At 18 years³ | -0.41 (-1.73, 0.91) | -0.55 (-1.63, 0.54) | | 0.672 | 0.997 |

^1^Results are expressed as change in IQ (points) associated with one z score of the conditional variable.

^2^ Adjusted for family income at birth, parental years of schooling, maternal skin color, maternal smoking during pregnancy, predominant breastfeeding duration. Birthweight was not adjusted for breastfeeding. IQ: Wechsler Adult Intelligence Scale.

³ Conditional z scores
